# [(2*R*,3*R*)-3-(4-Nitro­phen­yl)aziridin-2-yl]methanol monohydrate

**DOI:** 10.1107/S1600536813013391

**Published:** 2013-05-18

**Authors:** V. Gaumet, C. Denis, F. Leal, M. Madesclaire, V.P. Zaitsev

**Affiliations:** a UMR 990, INSERM, Université d’Auvergne, Laboratoire de Chimie Physique, Faculté de Pharmacie, 63001 Clermont-Ferrand, France; b Laboratoire de Chimie Thérapeutique, Faculté de Pharmacie, Université d’Auvergne, 63001 Clermont-Ferrand, France; c Samara State University, 433011 Samara, Russian Federation

## Abstract

The title monohydrate, C_9_H_10_N_2_O_3_·H_2_O, contains an aziridine ring including two contiguous stereocenters, both of which exhibit an *R* configuration. The methyl­hydroxy and nitro­phenyl groups are *cis*-disposed about the aziridine ring. The mean plane of the benzene ring is tilted to the aziridine ring by 66.65 (8)°. The nitro group is nearly coplanar with the benzene ring [dihedral angle = 2.5 (2)°]. In the crystal, the components are linked by N—H⋯O, O—H⋯N and O—H⋯O hydrogen bonds, generating supra­molecular layers parallel to (001).

## Related literature

For the biological activity of aziridine derivatives, see: Li *et al.* (1995 ▶); Sheldon *et al.* (1999 ▶); Danshiitsoodol *et al.* (2006 ▶); Vicik *et al.* (2006 ▶); Keniche *et al.* (2011 ▶); Lee *et al.* (1992 ▶); Ngo *et al.* (1998 ▶). For the use of chiral aziridines as precursors for pharmaceutical products, see: Kim *et al.* (2001 ▶). For related structures, see: Zhu *et al.* (2006 ▶). For details of the synthesis, see: Madesclaire *et al.* (2013 ▶). For determination of the absolute configuration, see: Hooft *et al.* (2008 ▶).
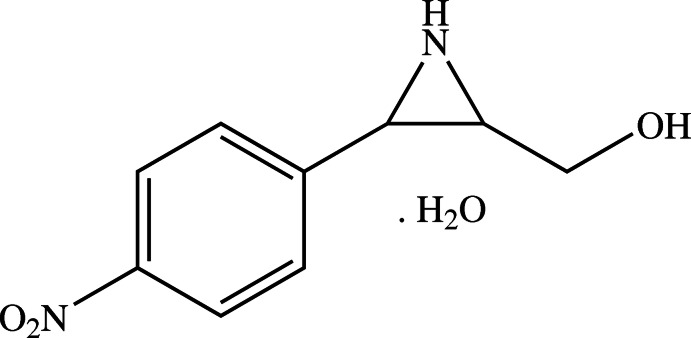


## Experimental

### 

#### Crystal data

C_9_H_10_N_2_O_3_·H_2_O
*M* *_r_* = 212.21
Monoclinic, 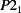

*a* = 6.3064 (2) Å
*b* = 5.4695 (2) Å
*c* = 14.6481 (5) Å
β = 94.303 (2)°
*V* = 503.83 (3) Å^3^
*Z* = 2
Mo *K*α radiation
μ = 0.11 mm^−1^
*T* = 296 K
0.68 × 0.44 × 0.06 mm

#### Data collection

Bruker APEXII CCD diffractometer
Absorption correction: multi-scan (*SADABS*; Bruker, 2012 ▶) *T*_min_ = 0.944, *T*_max_ = 1.000
5357 measured reflections
2383 independent reflections
2031 reflections with *I* > 2σ(*I*)
*R*_int_ = 0.020

#### Refinement

*R*[*F* ^2^ > 2σ(*F* ^2^)] = 0.035
*wR*(*F* ^2^) = 0.086
*S* = 1.04
2383 reflections
152 parameters
2 restraints
H atoms treated by a mixture of independent and constrained refinement
Δρ_max_ = 0.13 e Å^−3^
Δρ_min_ = −0.19 e Å^−3^

### 

Data collection: *APEX2* (Bruker, 2012 ▶); cell refinement: *SAINT* (Bruker, 2012 ▶); data reduction: *SAINT*; program(s) used to solve structure: *SHELXS97* (Sheldrick, 2008 ▶); program(s) used to refine structure: *SHELXL97* (Sheldrick, 2008 ▶); molecular graphics: *ORTEP-3 for Windows* (Farrugia, 2012 ▶) and *PLATON* (Spek, 2009 ▶); software used to prepare material for publication: *publCIF* (Westrip, 2010 ▶).

## Supplementary Material

Crystal structure: contains datablock(s) global, I. DOI: 10.1107/S1600536813013391/kp2453sup1.cif

Structure factors: contains datablock(s) I. DOI: 10.1107/S1600536813013391/kp2453Isup2.hkl

Supplementary material file. DOI: 10.1107/S1600536813013391/kp2453Isup3.cml

Additional supplementary materials:  crystallographic information; 3D view; checkCIF report

## Figures and Tables

**Table 1:** Hydrogen-bond geometry (Å, °)

*D*—H⋯*A*	*D*—H	H⋯*A*	*D*⋯*A*	*D*—H⋯*A*
N1—H1⋯O5^i^	0.91 (2)	2.24 (2)	3.064 (2)	150.0 (19)
O5—H5⋯O1*W* ^ii^	0.798 (19)	2.03 (2)	2.8303 (19)	175.0 (17)
O1*W*—H1*W*⋯N1	0.90 (2)	1.89 (2)	2.772 (2)	167 (2)
O1*W*—H2*W*⋯O1*W* ^iii^	0.90 (2)	2.00 (2)	2.8971 (9)	172 (3)

Symmetry codes: (i) 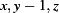; (ii) 
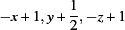
; (iii) 
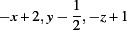
.

**Fig. 1.**
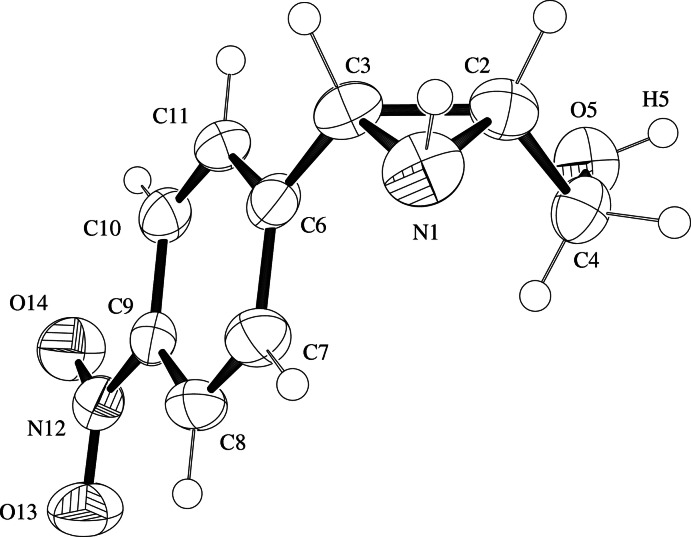
Molecular structure showing the atom-labelling scheme. Displacement ellipsoids are drawn at the 50% probability level. H atoms are represented by spheres of arbitrary radius.

**Fig. 2.**
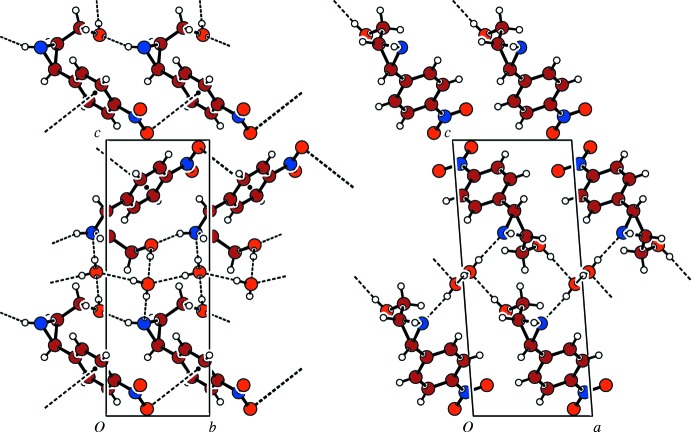
Structure projections for the title compound. Dashed lines represent intermolecular H-bond and nitro-group···π interactions. Left: projection along a, the centroids Cg are denoted by small black spheres. Right: projection along b, intra-chain interactions have been omited for clarity.

**Fig. 3.**
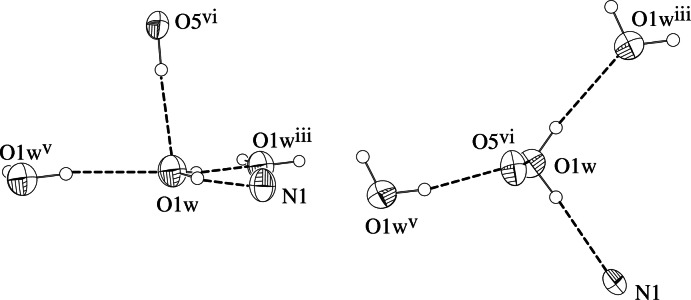
Two orthogonal views of hydrogen bonding around water oxygen atom O1w. Thermal ellipsoids are drawn at the 50% probability level. [Symmetry codes: (iii) -x + 2, y - 1/2, -z + 1; (v) -x + 2, y + 1/2, -z + 1; (vi) -x + 1, y - 1/2, -z + 1]

## supplementary crystallographic information

### Comment

Because of their powerful alkylating properties (Li *et al.*, 1995), aziridine-containing natural products such as mitomycin C, dibenzyl aziridine-2,3-dicarboxylate, *N*-(diethylphosphonopropionyl)-2-hydroxymethylaziridine, aziridinylbenzoquinones are potent antitumor and antibiotic agents (Sheldon *et al.*, 1999; Danshiitsoodol *et al.*,2006; Vicik *et al.*, 2006; Keniche *et al.*, 2011; Lee *et al.*,1992; Ngo *et al.*, 1998). Furthermore chiral aziridine derivatives offer a combination of reactivity, synthetic flexibility, and atom economy that is commonly employed in heterocyclic chemistry for the preparation of important pharmaceutical products like effective antiretroviral drugs used in the treatment of the human immunodeficiency virus (Kim *et al.*, 2001).

Dimensions of aziridine cycles highlight a pronounced C—C bond shortening [1.487 (2) Å] and a little C—N bond lengthening [1.478 (2) Å on average] when compared to normal open chain C—C (1.54 Å) and C—N (1.46 Å) bond lengths. Aziridine endocyclic angles are close to 60° [59.7 (1) - 60.4 (1)°] and the geometry at nitrogen is pyramidal (Zhu *et al.*, 2006). Methyl hydroxyl and nitrophenyl groups are *cis*-disposed about the aziridine ring (Fig. 1) and only one invertomer was found that has the NH proton on the least hindered ring side. The nitro group is nearly coplanar with the benzene ring [C8—C9—N12—O13 = 2.5 (2)°]. The mean N—O bond lengths are in an usual range [1.216 (2) - 1.223 (2) Å] for aromatic nitro groups.

C_9_H_10_N_2_O_3_ molecules are linked into infinite chains *via* N1—H1···O5 intermolecular hydrogen bonds (Table 1). Along these chains, the nitro phenyl groups are located on the same side in an isotactic way (Fig. 2). Accordingly, the chain structure is additionally stabilized by an intermolecular NO_2_···π interaction, namely N12—O14···C_g_^iv^ [symmetry code: iv: *x*, *y* + 1, *z*], with distance of 3.539 (1) Å between O14 atom and centroid, C_g_, of (C6—C11) aromatic ring. Chains are running parallel to the [0 1 0] direction and define hydrophilic channels occupied by water molecules.The water oxygen atom O1w acts as both donor and acceptor in four hydrogen bonds. The dihedral angle between the acceptor plane (defined by O1w^v^, O1w and O5^vi^) and the donor plane (defined by O1w^iii^, O1w and N1) is 86.67 (4)°. O1w^v^ lies 0,216 (6) Å below the donor plane (Fig. 3). The hydrogen bonds lie in the lone-pair plane of the O1w atom but no preference could be discerned for the *sp*^3^ (*i.e.* tetrahedral) lone pair directions within that plane. The strong H-bond network between chains and water molecules builds up [(C_9_H_10_N_2_O_3_)_2_(H_2_O)_2_]_∞_ layers parallel to the (0 0 1) plane (Fig. 2).

### Experimental

(2*R*,3*R*)-3-(4-Nitrophenyl)aziridin-2-yl]methanol was synthesized from (1*S*,2*S*)-2-amino-1-(4-nitrophenyl)-1,3-propandiol by the four-step method developped by Madesclaire *et al.* (2013). Plate and colourless crystals of the title coumpund were obtained by slow evaporation in air of a 1:10 (*v/v*) methanol-ethyl acetate solution. The formation of the monohydrated compound is certainly favored by the high hygroscopicity of methanol which absorbs moisture from the air. Once formed, monocrystals are stable in air for several weeks.

### Refinement

All C-bound H atoms were positioned geometrically [C—H = 0.97 Å for methanediyl, C—H = 0.98 Å for methanetriyl and C—H = 0.93 Å for aromatic H atoms] and included in the refinement in the riding-model approximation with *U*_iso_(H) = 1.2*U*_eq_(C). The N– and O–bound H atoms were located from difference-Fourier maps and refined freely. O—H distances in water molecule were restrained to be equal with standard deviation 0.02, the actual value of these distances was free to refine. The absolute structure was set by reference to the known chirality of the carbon atom bearing the hydroxymethyl group, namely C2 (note that the Cahn-Ingold-Prelog designation at C2 atom is reversed by comparison with that of the starting material due to the change in priority of the substituents). Hooft parameter is poorly defined 0.1 (6), however examination of the Bijvoet pairs using the likelihood methods (Hooft *et al.*, 2008) confirms that the absolute configuration had been correctly assigned with probability higher than 0.816.

### Crystal data

**Table:**

C_9_H_10_N_2_O_3_·H_2_O	*F*(000) = 224
*M**_r_* = 212.21	*D*_x_ = 1.399 Mg m^−^^3^
Monoclinic, *P*2_1_	Melting point: 379 K
Hall symbol: P 2yb	Mo *K*α radiation, λ = 0.71073 Å
*a* = 6.3064 (2) Å	Cell parameters from 98 reflections
*b* = 5.4695 (2) Å	θ = 5.1–26.3°
*c* = 14.6481 (5) Å	µ = 0.11 mm^−^^1^
β = 94.303 (2)°	*T* = 296 K
*V* = 503.83 (3) Å^3^	Plate, colourless
*Z* = 2	0.68 × 0.44 × 0.06 mm

### Data collection

**Table:**

Bruker APEXII CCD diffractometer	2383 independent reflections
Radiation source: fine-focus sealed tube	2031 reflections with *I* > 2σ(*I*)
Graphite monochromator	*R*_int_ = 0.020
φ and ω scans	θ_max_ = 29.1°, θ_min_ = 4.0°
Absorption correction: multi-scan (*SADABS*; Bruker, 2012)	*h* = −8→7
*T*_min_ = 0.944, *T*_max_ = 1.000	*k* = −7→6
5357 measured reflections	*l* = −20→19

### Refinement

**Table:**

Refinement on *F*^2^	Secondary atom site location: difference Fourier map
Least-squares matrix: full	Hydrogen site location: inferred from neighbouring sites
*R*[*F*^2^ > 2σ(*F*^2^)] = 0.035	H atoms treated by a mixture of independent and constrained refinement
*wR*(*F*^2^) = 0.086	*w* = 1/[σ^2^(*F*_o_^2^) + (0.0435*P*)^2^ + 0.0307*P*] where *P* = (*F*_o_^2^ + 2*F*_c_^2^)/3
*S* = 1.04	(Δ/σ)_max_ < 0.001
2383 reflections	Δρ_max_ = 0.13 e Å^−^^3^
152 parameters	Δρ_min_ = −0.19 e Å^−^^3^
2 restraints	Absolute structure: The absolute configuration was assigned to agree with that of its precusor at the chiral center C2.
Primary atom site location: structure-invariant direct methods	

### Special details

**Table:**

Geometry. All e.s.d.'s (except the e.s.d. in the dihedral angle between two l.s. planes) are estimated using the full covariance matrix. The cell e.s.d.'s are taken into account individually in the estimation of e.s.d.'s in distances, angles and torsion angles; correlations between e.s.d.'s in cell parameters are only used when they are defined by crystal symmetry. An approximate (isotropic) treatment of cell e.s.d.'s is used for estimating e.s.d.'s involving l.s. planes.
Refinement. Refinement of *F*^2^ against ALL reflections. The weighted *R*-factor *wR* and goodness of fit *S* are based on *F*^2^, conventional *R*-factors *R* are based on *F*, with *F* set to zero for negative *F*^2^. The threshold expression of *F*^2^ > σ(*F*^2^) is used only for calculating *R*-factors(gt) *etc*. and is not relevant to the choice of reflections for refinement. *R*-factors based on *F*^2^ are statistically about twice as large as those based on *F*, and *R*- factors based on ALL data will be even larger.

### Fractional atomic coordinates and isotropic or equivalent isotropic displacement parameters (Å^2^)

**Table:**

	*x*	*y*	*z*	*U*_iso_*/*U*_eq_	
N1	0.6266 (2)	0.3667 (2)	0.33556 (10)	0.0457 (3)	
H1	0.573 (3)	0.212 (5)	0.3340 (14)	0.063 (6)*	
C2	0.4417 (2)	0.5260 (3)	0.34835 (10)	0.0394 (3)	
H2	0.3069	0.4423	0.3563	0.047*	
C3	0.5261 (2)	0.5024 (3)	0.25666 (9)	0.0394 (3)	
H3	0.4388	0.4018	0.2131	0.047*	
C4	0.4863 (3)	0.7468 (3)	0.40646 (10)	0.0397 (3)	
H4A	0.4745	0.7051	0.4702	0.048*	
H4B	0.6303	0.8024	0.3998	0.048*	
O5	0.3410 (2)	0.9368 (2)	0.38063 (8)	0.0489 (3)	
H5	0.256 (3)	0.927 (4)	0.4181 (12)	0.051 (5)*	
C6	0.6449 (2)	0.7016 (3)	0.21394 (9)	0.0357 (3)	
C7	0.8526 (2)	0.7649 (3)	0.24213 (10)	0.0426 (4)	
H7	0.9250	0.6782	0.2894	0.051*	
C8	0.9530 (2)	0.9552 (3)	0.20089 (9)	0.0422 (4)	
H8	1.0915	0.9990	0.2205	0.051*	
C9	0.8439 (2)	1.0790 (3)	0.13006 (9)	0.0361 (3)	
C10	0.6386 (2)	1.0188 (3)	0.09979 (9)	0.0408 (4)	
H10	0.5676	1.1043	0.0519	0.049*	
C11	0.5409 (2)	0.8300 (3)	0.14185 (9)	0.0401 (4)	
H11	0.4025	0.7871	0.1218	0.048*	
N12	0.9485 (2)	1.2823 (3)	0.08617 (8)	0.0438 (3)	
O13	1.13280 (19)	1.3298 (3)	0.11207 (9)	0.0642 (4)	
O14	0.8479 (2)	1.3952 (3)	0.02591 (8)	0.0611 (4)	
O1W	0.9400 (2)	0.4031 (3)	0.47803 (9)	0.0568 (3)	
H1W	0.856 (4)	0.392 (5)	0.4262 (14)	0.089 (8)*	
H2W	0.990 (4)	0.252 (4)	0.491 (2)	0.092 (8)*	

### Atomic displacement parameters (Å^2^)

**Table:**

	*U*^11^	*U*^22^	*U*^33^	*U*^12^	*U*^13^	*U*^23^
N1	0.0548 (8)	0.0273 (7)	0.0541 (8)	0.0025 (6)	−0.0019 (6)	0.0015 (6)
C2	0.0401 (8)	0.0322 (8)	0.0459 (7)	−0.0037 (6)	0.0026 (6)	0.0051 (7)
C3	0.0425 (8)	0.0338 (8)	0.0408 (7)	−0.0033 (7)	−0.0047 (6)	−0.0053 (6)
C4	0.0506 (9)	0.0344 (8)	0.0341 (7)	0.0016 (7)	0.0025 (6)	0.0029 (6)
O5	0.0594 (7)	0.0371 (6)	0.0511 (6)	0.0079 (6)	0.0090 (6)	0.0079 (6)
C6	0.0374 (7)	0.0395 (8)	0.0298 (6)	0.0015 (6)	0.0000 (5)	−0.0076 (6)
C7	0.0364 (7)	0.0519 (10)	0.0384 (7)	0.0040 (7)	−0.0054 (6)	0.0057 (8)
C8	0.0305 (7)	0.0553 (10)	0.0399 (7)	−0.0006 (7)	−0.0035 (5)	0.0013 (8)
C9	0.0368 (7)	0.0430 (8)	0.0288 (6)	0.0017 (6)	0.0048 (5)	−0.0038 (6)
C10	0.0390 (8)	0.0537 (10)	0.0288 (6)	0.0047 (7)	−0.0030 (5)	0.0015 (7)
C11	0.0325 (7)	0.0546 (10)	0.0322 (7)	−0.0012 (7)	−0.0044 (5)	−0.0036 (7)
N12	0.0453 (7)	0.0488 (9)	0.0373 (6)	−0.0046 (6)	0.0044 (5)	−0.0043 (6)
O13	0.0530 (7)	0.0721 (9)	0.0658 (8)	−0.0226 (7)	−0.0060 (6)	0.0051 (7)
O14	0.0650 (8)	0.0656 (9)	0.0518 (6)	−0.0047 (7)	−0.0025 (5)	0.0195 (6)
O1W	0.0527 (7)	0.0553 (8)	0.0612 (7)	−0.0004 (6)	−0.0036 (6)	−0.0010 (7)

### Geometric parameters (Å, º)

**Table:**

N1—C3	1.476 (2)	C7—C8	1.381 (2)
N1—C2	1.479 (2)	C7—H7	0.9300
N1—H1	0.91 (2)	C8—C9	1.379 (2)
C2—C3	1.487 (2)	C8—H8	0.9300
C2—C4	1.492 (2)	C9—C10	1.377 (2)
C2—H2	0.9800	C9—N12	1.466 (2)
C3—C6	1.487 (2)	C10—C11	1.372 (2)
C3—H3	0.9800	C10—H10	0.9300
C4—O5	1.4181 (19)	C11—H11	0.9300
C4—H4A	0.9700	N12—O14	1.2161 (17)
C4—H4B	0.9700	N12—O13	1.2231 (16)
O5—H5	0.798 (19)	O1W—H1W	0.895 (19)
C6—C7	1.387 (2)	O1W—H2W	0.90 (2)
C6—C11	1.3907 (19)		
			
C3—N1—C2	60.44 (10)	C7—C6—C11	118.73 (14)
C3—N1—H1	108.1 (13)	C7—C6—C3	123.49 (13)
C2—N1—H1	104.8 (14)	C11—C6—C3	117.77 (13)
N1—C2—C3	59.67 (10)	C8—C7—C6	120.79 (13)
N1—C2—C4	115.62 (13)	C8—C7—H7	119.6
C3—C2—C4	121.37 (12)	C6—C7—H7	119.6
N1—C2—H2	116.0	C9—C8—C7	118.67 (13)
C3—C2—H2	116.0	C9—C8—H8	120.7
C4—C2—H2	116.0	C7—C8—H8	120.7
N1—C3—C6	119.88 (13)	C10—C9—C8	121.95 (14)
N1—C3—C2	59.88 (10)	C10—C9—N12	118.91 (13)
C6—C3—C2	122.85 (12)	C8—C9—N12	119.14 (13)
N1—C3—H3	114.5	C11—C10—C9	118.58 (13)
C6—C3—H3	114.5	C11—C10—H10	120.7
C2—C3—H3	114.5	C9—C10—H10	120.7
O5—C4—C2	110.52 (12)	C10—C11—C6	121.27 (13)
O5—C4—H4A	109.5	C10—C11—H11	119.4
C2—C4—H4A	109.5	C6—C11—H11	119.4
O5—C4—H4B	109.5	O14—N12—O13	123.31 (15)
C2—C4—H4B	109.5	O14—N12—C9	118.43 (13)
H4A—C4—H4B	108.1	O13—N12—C9	118.25 (13)
C4—O5—H5	102.8 (15)	H1W—O1W—H2W	107 (2)
			
C3—N1—C2—C4	−112.85 (14)	C6—C7—C8—C9	−1.0 (2)
C2—N1—C3—C6	112.95 (15)	C7—C8—C9—C10	0.2 (2)
C4—C2—C3—N1	103.31 (15)	C7—C8—C9—N12	179.60 (14)
N1—C2—C3—C6	−108.12 (15)	C8—C9—C10—C11	0.1 (2)
C4—C2—C3—C6	−4.8 (2)	N12—C9—C10—C11	−179.30 (13)
N1—C2—C4—O5	152.10 (12)	C9—C10—C11—C6	0.4 (2)
C3—C2—C4—O5	83.41 (17)	C7—C6—C11—C10	−1.1 (2)
N1—C3—C6—C7	2.3 (2)	C3—C6—C11—C10	178.98 (14)
C2—C3—C6—C7	73.8 (2)	C10—C9—N12—O14	1.9 (2)
N1—C3—C6—C11	−177.77 (13)	C8—C9—N12—O14	−177.50 (14)
C2—C3—C6—C11	−106.31 (16)	C10—C9—N12—O13	−178.12 (14)
C11—C6—C7—C8	1.4 (2)	C8—C9—N12—O13	2.5 (2)
C3—C6—C7—C8	−178.68 (14)	C2—C4—O5—H5	99.5 (15)

### Hydrogen-bond geometry (Å, º)

**Table:**

*D*—H···*A*	*D*—H	H···*A*	*D*···*A*	*D*—H···*A*
N1—H1···O5^i^	0.91 (2)	2.24 (2)	3.064 (2)	150.0 (19)
O5—H5···O1*W*^ii^	0.798 (19)	2.03 (2)	2.8303 (19)	175.0 (17)
O1*W*—H1*W*···N1	0.90 (2)	1.89 (2)	2.772 (2)	167 (2)
O1*W*—H2*W*···O1*W*^iii^	0.90 (2)	2.00 (2)	2.8971 (9)	172 (3)

Symmetry codes: (i) *x*, *y*−1, *z*; (ii) −*x*+1, *y*+1/2, −*z*+1; (iii) −*x*+2, *y*−1/2, −*z*+1.
